# Challenges in Brugada Syndrome Stratification: Investigating *SCN5A* Mutation Localization and Clinical Phenotypes

**DOI:** 10.3390/ijms242316658

**Published:** 2023-11-23

**Authors:** Adriana Tarantino, Giuseppe Ciconte, Andrea Ghiroldi, Flavio Mastrocinque, Emanuele Micaglio, Antonio Boccellino, Gabriele Negro, Marco Piccoli, Federica Cirillo, Gabriele Vicedomini, Vincenzo Santinelli, Luigi Anastasia, Carlo Pappone

**Affiliations:** 1 Institute for Molecular and Translational Cardiology (IMTC), IRCCS Policlinico San Donato, Piazza Malan 2, San Donato Milanese, 20097 Milan, Italy; tarantino.adriana@hsr.it (A.T.); marco.piccoli@grupposandonato.it (M.P.); federica.cirillo@grupposandonato.it (F.C.); 2 School of Medicine, University Vita-Salute San Raffaele, Via Olgettina 58, 20132 Milan, Italy; giuseppe.ciconte@grupposandonato.it; 3 Arrhythmology Department, IRCCS Policlinico San Donato, Piazza Malan 2, San Donato Milanese, 20097 Milan, Italy; flavio.mastrocinque@grupposandonato.it (F.M.); emanuele.micaglio@grupposandonato.it (E.M.); antonio.boccellino@grupposandonato.it (A.B.); gabriele.negro@grupposandonato.it (G.N.); gabriele.vicedomini@grupposandoanto.it (G.V.); vincenzo.santinelli@grupposandonato.it (V.S.)

**Keywords:** voltage-gated sodium channel, protein unstructured regions, BrS genetics, PTMs in IDRs, *SCN5A* mutations, in silico prediction tools

## Abstract

Brugada Syndrome (BrS) is a genetic heart condition linked to sudden cardiac death. Though the *SCN5A* gene is primarily associated with BrS, there is a lack of comprehensive studies exploring the connection between *SCN5A* mutation locations and the clinical presentations of the syndrome. This study aimed to address this gap and gain further understanding of the syndrome. The investigation classified 36 high-risk BrS patients based on *SCN5A* mutations within the transmembrane/structured (TD) and intra-domain loops (IDLs) lacking a 3D structure. We characterized the intrinsically disordered regions (IDRs) abundant in IDLs, using bioinformatics tools to predict IDRs and post-translational modifications (PTMs) in NaV1.5. Interestingly, it was found that current predictive tools often underestimate the impacts of mutations in IDLs and disordered regions. Moreover, patients with *SCN5A* mutations confined to IDL regions—previously deemed ‘benign’—displayed clinical symptoms similar to those carrying ‘damaging’ variants. Our research illuminates the difficulty in stratifying patients based on *SCN5A* mutation locations, emphasizing the vital role of IDLs in the NaV1.5 channel’s functioning and protein interactions. We advocate for caution when using predictive tools for mutation evaluation in these regions and call for the development of improved strategies in accurately assessing BrS risk

## 1. Introduction

Brugada Syndrome (BrS) is an inherited arrhythmogenic disorder marked by an elevated risk of sudden cardiac death (SCD) [1]. This genetic channelopathy follows an autosomal dominant inheritance pattern with incomplete penetrance [2]. While research has unveiled its polygenic nature [3,4], *SCN5A* remains the only clinically associated gene commonly evaluated, although it is mutated in only approximately 20% of cases [5] affecting protein activity, synthesis, or processing and trafficking [6]. The *SCN5A* gene, encoding the alpha subunit of the cardiac sodium channel NaV1.5 [7], has a pivotal role in the rapid increase in cardiac action potential with the rapid influx of sodium ions (Na^+^) and initiates the cascades involved in excitation–contraction coupling within cardiomyocytes important for cardiac homeostasis [8]. Structurally, the NaV1.5 channel has conserved domains (TDs) interspersed with non-conserved intra-domain loops (IDLs). In particular, it comprises four homologous domains (D1–D4), each with six TDs, three IDLs (IDL1–3), and extensive intracellular N and C terminals [9]. IDL regions are highly flexible, lack proper secondary structure as a/b helices, and are enriched in Intrinsically Disordered Regions (IDR), which play critical roles in various biological functions such as gene regulation [10], cell cycle control [11], and signal transduction [12,13]. In transmembrane proteins, such as ion channels, IDRs are mostly prevalent in cytoplasmatic portions (IDLs) where the disordered score is three times higher than the external counterpart [14]. IDRs are characterized by high net charge, low hydrophobicity, a propensity to form adaptable coils, and low sequence complexity [15]. They contain linear motifs, molecular recognition features (MoRFs), post-translational modification (PTM) sites, and a peptide sequence that is rich in proline (P), glutamic acid (E), serine (S), and threonine (T) (PEST) motifs, which are crucial for encoding and decoding information for protein functions [16,17] and macromolecular interactions, including protein–protein, protein–DNA, and protein–RNA interactions [18,19]. Over 500 *SCN5A* gene variants, mainly localized in the pore/selectivity filter and transmembrane domain, are associated with BrS [7]. Nevertheless, their clinical interpretation largely depends on the use of predictive algorithms that often fail to match the disease severity observed in the clinic [20] or the loss of function observed in in vitro studies [10].

In light of these challenges, we aim to elucidate the relationship between the severity of BrS and the topological locations of *SCN5A* gene variants. Moreover, we intend to evaluate the performance of existing in silico prediction algorithms to help clinicians assess the impact of mutations in determining disease severity in patients at high clinical risk for BrS. Lately, we characterized IDL regions of the NaV1.5 channel with particular attention on IDRs.

## 2. Results

### 2.1. SCN5A Mutation Analysis on TD and IDL Segments in BrS Patients at High Risk of SCD

**Figure 1:**
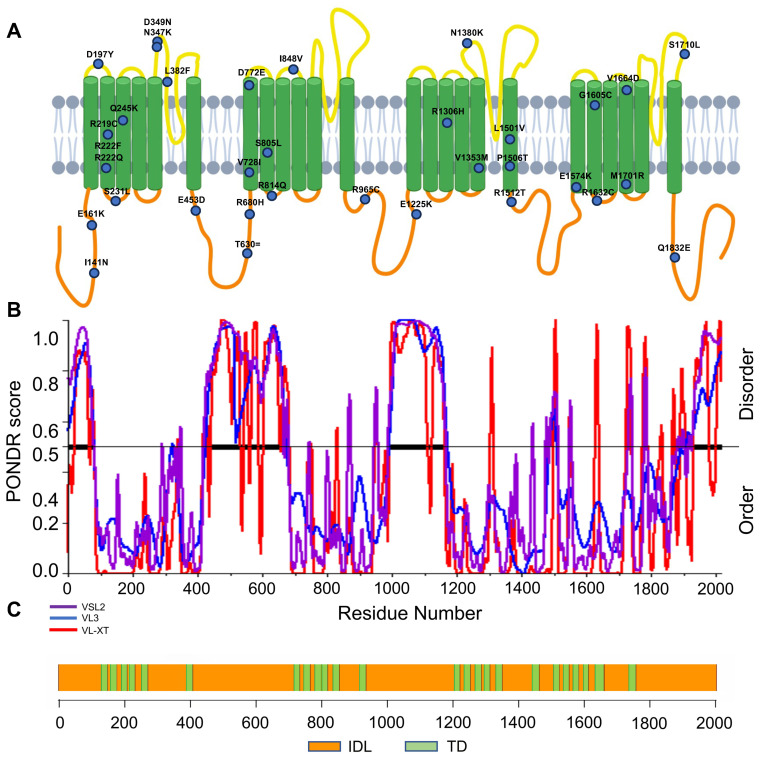
NaV1.5 is the most disordered member of the voltage-gated sodium channel family. (**A**) Localization of mutations in NaV1.5 in BrS cohort. (**B**) Disorder analysis of NaV1.5: PONDR-VLXT, PONDR-VSL2B, and PONDR-VL3 prediction tools were used to determine the disorder score of NaV1.5. Any value above 0.5 indicates intrinsic disorder. (**C**) Graphical illustration of NaV1.5 structure with TDs and IDL domains.

The 36 high-risk BrS patients were categorized based on the presence of mutations in TDs or IDL regions (Table 1; Figure 1A). Among them, 9 patients had mutations in the IDL regions and 27 in the TDs. Mutations occurring in the IDL domains were categorized as ‘*benign*’ by in silico tools at a significantly higher rate compared to mutations in TDs (44% vs. 15%) (Table 1). At a clinical level, 22% of patients with IDL mutations had documented appropriate implantable cardioverter defibrillator (ICD) therapies for ventricular arrhythmias, 10% had experienced an aborted cardiac arrest, 77% reported syncope, 33% exhibited a spontaneous type 1 pattern, and 44% had a family history of sudden death. In BrS patients with mutations in the TDs, 48% had documented appropriate ICD therapy for ventricular arrhythmias, 18% had experienced aborted cardiac arrest, 40% reported syncope, 25% exhibited a spontaneous type 1 pattern, and 22% had a family history of sudden death. However, patients with IDL mutations had significant atrial fibrillation compared to those carrying TDs. Table 1 also includes the filtered QRS duration (f-QRSd), root mean square voltage of the terminal 40 ms of the filtered QRS complex (RMS40), duration of low-amplitude signal < 40 µV (LAS40) values, and mapping of substrate size and potential duration (PD) at baseline and after ajmaline administration (Table 1 and Supplementary Table S1). Supplementary Tables S2 and S3 delineate the clinical phenotypes of BrS patients further categorized in the IDLs and TDs based on predictions of being ‘benign’ and ‘damaging’. Notability, there were no significant variations observed in clinical features when mutations were categorized based on their predicted pathogenicity, both in TD and in IDL.

### 2.2. Characterization of IDRs within IDLs in the NaV1.5 Channel

To characterize IDL domains in the NaV1.5 channel, we compared cryoEM data of the NaV1.5 protein with the in silico prediction of disordered protein regions using three programs: PONDR-VLXT, PONDR-VSL2B, and PONDR-VL3 (Figure 1B,C).

This analysis predicted 25 putative IDRs, including four fragments with more than 30 amino acids that have a consistent pattern across all members of the NaV channel family (amino acid positions IDR1 20–66, IDR2 441–532, IDR3 993–1111, 1125–1162, and IDR4 1932-1980) (Supplementary Figures S1 and S2), and phased in the IDL domains. The family of voltage-gated sodium channels includes nine members (NaV1.1–NaV1.9), with NaV1.5 being the longest. We calculated the disorder propensity degree for each family member and found that NaV1.5 had the highest average prediction score (0.328), the highest number of disordered amino acids (637), and the highest percentage of IDRs in the total protein length (31.6%) (Table 2).

The average overall sequence homology of NaV1.5 with the other eight members is 71.41%. Comparison of a conserved transmembrane domain (e.g., D2) increases the homology percentage to 80.82%, whereas comparison of the four longest IDRs yields mean homology values of 43.18%, 52.00%, 51.46%, and 35.71% for IDR1, IDR2, IDR3, and IDR4, respectively (Table 3 and Supplementary Figure S2).

### 2.3. PTM Analysis

**Figure 2:**
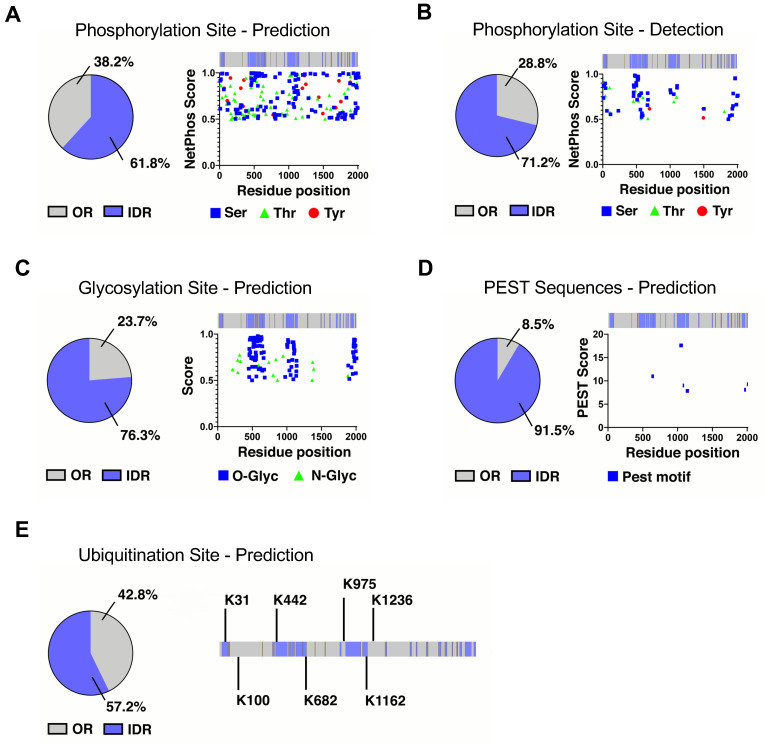
NaV1.5 PTM sites along the NaV1.5 protein channel. Percentage of ordered (OR) and disordered (IDR) regions in NaV1.5 (**A**) predicted to be phosphorylated; (**B**) demonstrated to be phosphorylated; (**C**) predicted to be N- and O-glycosylated; (**D**) predicted to be PEST motif; (**E**) predicted to be ubiquitinated.

We determined the putative sites for PTMs along the NaV1.5 protein and we found that the majority of them are in IDL domains, mostly in the four longest disordered regions (amino acid positions 20–66, 441–532, 993–1111–1125–1162, and 1932–1980). Overall, prediction of phosphorylation sites identified 38.2% of residues in ordered regions and 61.8% in IDRs (Figure 2A). Experimentally detected phosphorylated protein residues are annotated in PhosphositePlus. For NaV1.5, there are a total of 73 sites, of which 71.2% are in IDRs and 28.8% in ORs (Figure 2B). Potential glycosylation sites were predicted and yielded 114 residues: 76.3% in IDRs and 23.7% in ORs (Figure 2C). PEST sequences involved in protein degradation signaling were 91.5% in IDRs and 8.5% in ORs (Figure 2D). Finally, the prediction of ubiquitination sites identified seven candidates: three in ORs (42.8%) and four in IDRs (57.2%) (Figure 2E).

## 3. Discussion

In this work, we show that the mere presence of a mutation on the *SCN5A* gene, rather than its precise location within the sodium channel, is a determining factor in the development of BrS. Consistent with previous studies [21], our analysis shows a higher incidence of mutations in the *SCN5A* gene affecting the transmembrane and pore domains. However, contrary to expectations, the study revealed that the specific location of NaV1.5 mutations did not lead to significant differences in the clinical presentation of BrS. This result challenges classifications by in silico prediction tools that predominantly classified mutations in IDLs domain as ‘*benign*’. Furthermore, these findings have important implications for current clinical practice, as patients carrying *SCN5A* mutations in unstructured portions should not be rashly assured of a benign phenotype. Instead, it appears critical to perform further investigations to ensure appropriate clinical management. The effectiveness of correctly predicting the functional impact of a mutation is clearly influenced by its location, as the tools consider data from resolved protein structures and evolutionary conservation [22]. Indeed, using in silico characterization of IDL domains in the NaV1.5 channel, we found that they are enriched in intrinsically disordered regions (IDRs), which are well established to play a pivotal role in diverse aspects of protein functionality, such as recognition, regulation, and orchestration of numerous signaling events [23]. Considering that IDRs are tightly regulated regions within healthy cells, mutation accumulation in these areas can precipitate disease, as seen in the cases of *P53* [24] and the oncogene *PTEN* [25]. Although some recent studies have examined the IDR of transmembrane proteins and some channel types [19], we show here that NaV1.5 has the highest IDR among the nine homologous members of the voltage-gated sodium channel family. While structural similarity is maintained in the *core* of NaV, which represents the pore-forming or transmembrane domains, the length and positions of the IDR regions are specific to each IDL domain of the channel, suggesting their primary regulatory functions. In this context, PTMs play a central role in controlling the conformational dynamics of IDLs to interact with target proteins. As a result, the addition of different chemical groups leads to local and global conformational adjustments due to changes in the total energy of a protein [26,27]. In NaV1.5, phosphorylation and glycosylation make up the bulk of PTMs, arguing for their primary function in modulating the downstream channel cascade and ion flux through the pore channel by specific charges around the protein. Disruption of such essential sites in PTMs could lead to loss of flexibility and docking, ultimately limiting channel properties, as shown by Anthony et al. [28], where acquired arrhythmogenic phenotypes were observed after loss of PTMs.

However, most studies focus on mutations in folded regions and often neglect mutations in unstructured or disordered regions or even categorize them as variants of unknown significance, despite their common occurrence in disease-associated proteins [29,30,31]. As a result, prediction tools that assess the ‘pathogenicity’ of mutations by analyzing changes in the structural stability of proteins are often unable to make accurate predictions in disordered regions [29,32]. Indeed, our results provide an opportunity to reevaluate and explain previous observations documented in the literature. For instance, the Q1832E mutation, which was initially predicted to be ‘*benign*’ and located in the disordered regions of IDL domains, was not only identified in a patient with a severe BrS phenotype but also demonstrated a detrimental effect on channel function in previous in vitro functional studies [33,34]. Thus, it is conceivable that such mutations, which are mostly present in the external flexible loops of the channel, interfere with the interaction with key partner proteins of NaV1.5. This disruption could result in the failure to recognize certain residues from specific protein interactors, possibly contributing to BrS [35]. In our study, we have emphasized the importance of NaV1.5 within the cardiomyocyte network. We have uncovered the significant impact of mutations in often-overlooked unstructured regions. These mutations challenge existing prediction algorithms, highlighting the need for improved tools to assess them accurately. We also suggest a more comprehensive analysis of network changes, considering these mutations in unstructured regions, as they may have a substantial influence on the complex molecular network.

## 4. Materials and Methods

### 4.1. Study Population and Clinical Data

We conducted a retrospective analysis on a cohort of 36 unrelated BrS patients clinically judged to be at high risk for sudden cardiac death according to the latest guidelines [36], 9 of whom carried *SCN5A* missense mutations in IDLs and 27 in TD domains. Only patients with *SCN5A* mutations were considered; those with genetic mutations in other genes associated with BrS were excluded from the study. Additionally, those with comorbidities, particularly metabolic syndrome, coronary arterial conditions, and autoimmune diseases, were also excluded from the investigation. All patients met the diagnostic criteria for BrS, including a spontaneous or drug-induced type 1 Brugada ECG pattern. Clinical data, encompassing demographics, medical history, 12-lead ECG, and implantable cardioverter-defibrillator (ICD) outcomes, were collected from medical records. The study adhered to the Declaration of Helsinki, with informed consent obtained from all participants.

### 4.2. Genetic Analysis and Mutation Classification

All patients were studied using a Next Generation Sequencing panel of genes, including *SCN5A*, from peripheral blood-extracted DNA. The DNA was extracted from peripheral blood and treated to obtain libraries with a total content of 575 kb of genomic DNA, using 50 nanograms of DNA input quantity. The libraries were deep sequenced, and after removing duplicates and filtering low-quality reads, a mean target coverage of 106X was obtained. All Next Generation Sequencing data were confirmed using Sanger sequencing following the American College of Medical Genetics (ACMG) [37] guidelines, and the Sanger result was consistent with the Next Generation Sequencing output in all cases (100%). The prediction of missense mutations was obtained using three different tools: Sorting Intolerant from Tolerant (SIFT), Poly-phen2, and Clinvar.

### 4.3. Assessment of Arrhythmogenic Substrates

All enrolled patients underwent an electrophysiological study (EPS) and endo-epicardial mapping to assess the arrhythmogenic substrate. The extent of the arrhythmogenic substrate in the epicardium was quantified using established criteria. All patients underwent a combined endo-epicardial mapping procedure using a three-dimensional (3D) mapping system (CARTO 3, Biosense Webster, Irvine, CA, USA). All maps were obtained at baseline conditions and after the ajmaline test (up to 1 mg/kg over 10 min).

### 4.4. Prediction of Protein Disorder

All information about protein structures was obtained with cryoEM structure (PDB: 7DTC); disordered protein regions were predicted using a variety of computational tools tailored for different types of proteins and regions. The IUPRED algorithm was employed to predict disordered regions in proteins, while PONDR-VSL2B was utilized for proteins that contain a combination of structured and disordered regions. For proteins known to be fully disordered or those possessing long disordered regions, the PONDR-VL3 algorithm was applied. Lastly, to predict MoRFs, the PONDR-VLXT (Variously Long and X-ray Terminal) tool was utilized.

### 4.5. PTMs In Silico Prediction

We investigated PTMs such as phosphorylation, glycosylation, ubiquitination, and PEST sequences using prediction tools tailored for each modification. To estimate the phosphorylation of serine, threonine, and tyrosine residues, we used the NetPhos 3.1 Server (https://services.healthtech.dtu.dk/service.php?NetPhos-3.1) (accessed on 1 March 2023), considering scores above 0.5, and consulted PhosphositePlus [29] as well. To predict N- and O-linked glycosylation at asparagine (N), serine (S), and threonine (T) residues, we employed the GPP Prediction Server (https://comp.chem.nottingham.ac.uk/glyco/) (accessed on 1 March 2023). To identify lysine (K) ubiquitination, we utilized the PRmePRed tool (http://www.ubpred.org) (accessed on 1 March 2023). Lastly, to locate PEST sequences, we applied the ePEST algorithm (https://emboss.bioinformatics.nl/cgi-bin/emboss/epestfind) (accessed on 1 March 2023).

### 4.6. Statistical Analysis

The data were presented as mean ± standard deviation (SD). To assess the distribution of the data, a Shapiro–Wilk test was conducted. A chi-square test was utilized to determine the statistical significance of the results, using GraphPad Prism 9 software. A *p*-value of less than 0.05 was considered to indicate a statistically significant outcome.

## 5. Conclusions

In conclusion, our results support the notion that prediction tools may not be sufficient to accurately assess the pathogenicity of mutations, especially in IDLs. However, these regions, also enriched in IDRs, can disrupt the interaction between NaV1.5 and essential partner proteins once mutated, eventually hindering the proper recognition of specific residues by key protein interactors and contributing to the development of BrS.

This underscores the importance of developing more precise methods to assess the functional implications of mutations in these regions as they may have a substantial influence on the NaV1.5 molecular network.

## Figures and Tables

**Table 1:** Study population of patients with mutations in transmembrane domains (TDs) and intra-domain loops (IDLs). Clinical, anatomical, and electrophysiological characteristics of the 36 probands at high risk for sudden cardiac death.

	*SCN5A* Variants in TD Domains (*n* = 27; 75%)	*SCN5A* Variants in IDL Domains (*n* = 9; 25%)	*p* Value
Mutation prediction (benign%)	3 (15)	4 (44)	0.0286 *
Male, *n* (%)	20 (74)	4 (44)	0.1024
Age (years) (mean ± SD)	42.35 ± 12.08	39.77 ± 10.88	0.1772
Spontaneous type 1 pattern, *n* (%)	7 (25)	3 (33)	0.6674
Family history of sudden death, *n* (%)	6 (22)	4 (44)	0.1973
Aborted cardiac arrest, *n* (%)	5 (18)	1 (10)	0.6055
Syncope, *n* (%)	11 (40)	7 (77)	0.0026 *
Spontaneous VT/VF requiring ICD therapy, *n* (%)	13 (48)	2 (22)	0.1718
Atrial fibrillation, *n* (%)	9 (33)	6 (66)	0.0789
Atrial flutter, *n* (%)	1 (4)	0	0.4008
QRS duration ≥ 120 ms, *n* (%)	13 (48)	2 (22)	0.0865
f-QRSd (mean ± SD)	127.4 ± 17.85	107.9 ± 17.42	0.0099
RMS40 (mean ± SD)	15,482 ± 84.19	13,842 ± 10.33	0.3124
LAS40 (mean ± SD)	49.29 ± 18.65	46.67 ± 15.52	0.9160
Substrate size baseline (cm^2^)	9.042 ± 3.507	5.125 ± 4.824	0.0556
Potential duration baseline (ms)	133.0 ± 46.43	109.3 ± 37.51	0.2809
Substrate size after ajmaline (cm^2^)	20.29 ± 5.982	17.66 ± 9.131	0.5027
Potential duration after ajmaline (ms)	231.0 ± 27.67	214.5 ± 21.03	0.0991

Abbreviations: VT/VF: ventricular tachycardia and ventricular fibrillation. * *p* < 0.05

**Table 2:** IDR enrichment in the voltage-gated sodium channel family. Characteristics of nine voltage-gated sodium channel family members: length of the protein, number of IDR regions, average prediction score, number of total disordered amino acids considering the TOP-IDP score, percentage of IDRs.

Protein	Length (AA)	N.of IDR	Average Prediction Score	N.of Disordered AA	Percentage of IDR
**NaV1.1**	2009	29	0.275	517	25.73
**NaV1.2**	2005	25	0.276	538	26.83
**NaV1.3**	2000	23	0.268	520	26
**NaV1.4**	1836	19	0.253	437	23.80
**NaV1.5**	2016	25	0.328	637	31.60
**NaV1.6**	1980	24	0.268	504	25.45
**NaV1.7**	1988	29	0.282	538	27.08
**NaV1.8**	1956	25	0.272	498	25.46
**NaV1.9**	1791	21	0.219	330	18.43

**Table 3:** Identity between NaV1.5 and other NaV family members. Percentage of identity of IDRs among voltage-gated sodium channel family members for D2 (pos. 718–938), IDR1 (pos. 20–66); IDR2 (pos. 441–532); IDR3 (pos. 993–1111; 1125–1162); IDR4 (pos. 1932–1980).

NaV	% Identity	D2 (aa 718–938)	IDR1 (aa 20–66)	IDR2 (aa 441–532)	IDR3 (aa 993–1111; 1125–1162)	IDR4 (aa 1932–1980)
**NaV1.1**	71.17	80.82	44.19	62.18	48.49	47.06
**NaV1.2**	71.95	81.74	47.73	58.10	53.50	57.58
**NaV1.3**	72.09	83.11	47.73	72.38	52.75	50.00
**NaV1.4**	73.92	82.65	55.00	38.89	46.68	55.56
**NaV1.6**	71.41	80.82	43.18	52.00	51.46	35.71
**NaV1.7**	70.16	79.00	46.51	52.82	40.79	53.12
**NaV1.8**	71.96	76.64	51.16	46.75	53.00	56.25
**NaV1.9**	63.24	69.71	57.14	53.12	36.60	16.00

## Data Availability

The raw data supporting the conclusions of this manuscript will be made available by the authors, without undue reservation, to any qualified researcher.

## Supplementary Materials

The supporting information can be downloaded at: https://www.mdpi.com/article/10.3390/ijms242316658/s1.

## References

[1] Juang J.-M.J., Horie M. (2016). Genetics of Brugada syndrome. J. Arrhythmia.

[2] Quan X.Q., Li S., Liu R., Zheng K., Wu X.F., Tang Q. (2016). A meta-analytic review of prevalence for Brugada ECG patterns and the risk for death. Medicine.

[3] Monasky M.M., Micaglio E., Ciconte G., Pappone C. (2020). Brugada Syndrome: Oligogenic or Mendelian Disease?. Int. J. Mol. Sci..

[4] Hosseini S.M., Kim R., Udupa S., Costain G., Jobling R., Liston E., Jamal S.M., Szybowska M., Morel C.F., Bowdin S. (2018). Reappraisal of Reported Genes for Sudden Arrhythmic Death: Evidence-Based Evaluation of Gene Validity for Brugada Syndrome. Circulation.

[5] Sonoda K., Ohno S., Ozawa J., Hayano M., Hattori T., Kobori A., Yahata M., Aburadani I., Watanabe S., Matsumoto Y. (2018). Copy number variations of SCN5A in Brugada syndrome. Heart Rhythm..

[6] Jiang D., Shi H., Tonggu L., Gamal El-Din T.M., Lenaeus M.J., Zhao Y., Yoshioka C., Zheng N., Catterall W.A. (2020). Structure of the Cardiac Sodium Channel. Cell.

[7] Kapplinger J.D., Tester D.J., Alders M., Benito B., Berthet M., Brugada J., Brugada P., Fressart V., Guerchicoff A., Harris-Kerr C. (2010). An international compendium of mutations in the SCN5A-encoded cardiac sodium channel in patients referred for Brugada syndrome genetic testing. Heart Rhythm..

[8] Dong C., Wang Y., Ma A., Wang T. (2020). Life Cycle of the Cardiac Voltage-Gated Sodium Channel NaV1.5. Front. Physiol..

[9] Terjung R. (2011). Comprehensive Physiology.

[10] Colak R., Kim T., Michaut M., Sun M., Irimia M., Bellay J., Myers C.L., Blencowe B.J., Kim P.M. (2013). Distinct Types of Disorder in the Human Proteome: Functional Implications for Alternative Splicing. PLoS Comput. Biol..

[11] Das R.K., Huang Y., Phillips A.H., Kriwacki R.W., Pappu R.V. (2016). Cryptic sequence features within the disordered protein p27^Kip1^ regulate cell cycle signaling. Proc. Natl. Acad. Sci. USA.

[12] Basu S., Bahadur R.P. (2016). A structural perspective of RNA recognition by intrinsically disordered proteins. Cell Mol. Life Sci..

[13] Sammak S., Zinzalla G. (2015). Targeting protein–protein interactions (PPIs) of transcription factors: Challenges of intrinsically disordered proteins (IDPs) and regions (IDRs). Prog. Biophys. Mol. Biol..

[14] Minezaki Y., Homma K., Nishikawa K. (2007). Intrinsically disordered regions of human plasma membrane proteins preferentially occur in the cytoplasmic segment. J. Mol. Biol..

[15] Dunker A.K., Lawson J.D., Brown C.J., Williams R.M., Romero P., Oh J.S., Oldfield C.J., Campen A.M., Ratliff C.M., Hipps K.W. (2001). Intrinsically disordered protein. J. Mol. Graph. Model..

[16] Hsu W.-L., Oldfield C.J., Xue B., Meng J., Huang F., Romero P., Uversky V.N., Dunker A.K. (2013). Exploring the binding diversity of intrinsically disordered proteins involved in one-to-many binding: Exploring the Binding Diversity of IDPs. Protein Sci..

[17] Owen I., Shewmaker F. (2019). The Role of Post-Translational Modifications in the Phase Transitions of Intrinsically Disordered Proteins. Int. J. Mol. Sci..

[18] Vacic V., Iakoucheva L.M. (2012). Disease mutations in disordered regions—Exception to the rule?. Mol. BioSyst..

[19] Goretzki B., Guhl C., Tebbe F., Harder J.-M., Hellmich U.A. (2021). Unstructural Biology of TRP Ion Channels: The Role of Intrinsically Disordered Regions in Channel Function and Regulation. J. Mol. Biol..

[20] Iakoucheva L.M., Brown C.J., Lawson J.D., Obradović Z., Dunker A.K. (2002). Intrinsic Disorder in Cell-signaling and Cancer-associated Proteins. J. Mol. Biol..

[21] Ackerman M.J., Splawski I., Makielski J.C., Tester D.J., Will M.L., Timothy K.W., Keating M.T., Jones G., Chadha M., Burrow C.R. (2004). Spectrum and prevalence of cardiac sodium channel variants among black, white, Asian, and Hispanic individuals: Implications for arrhythmogenic susceptibility and Brugada/long QT syndrome genetic testing. Heart Rhythm..

[22] Ng P.C., Henikoff S. (2001). Predicting deleterious amino acid substitutions. Genome Res..

[23] Dunker A.K., Babu M.M., Barbar E., Blackledge M., Bondos S.E., Dosztányi Z., Dyson H.J., Forman-Kay J., Fuxreiter M., Gsponer J. (2013). What’s in a name? Why these proteins are intrinsically disordered: Why these proteins are intrinsically disordered. Intrinsically Disord. Proteins.

[24] Kirilyuk A., Shimoji M., Catania J., Sahu G., Pattabiraman N., Giordano A., Albanese C., Mocchetti I., Toretsky J.A., Uversky V.N. (2012). An Intrinsically Disordered Region of the Acetyltransferase p300 with Similarity to Prion-Like Domains Plays a Role in Aggregation. PLoS ONE.

[25] Malaney P., Pathak R.R., Xue B., Uversky V.N., Davé V. (2013). Intrinsic Disorder in PTEN and its Interactome Confers Structural Plasticity and Functional Versatility. Sci. Rep..

[26] Darling A.L., Uversky V.N. (2018). Intrinsic Disorder and Posttranslational Modifications: The Darker Side of the Biological Dark Matter. Front. Genet..

[27] Forman-Kay J.D., Mittag T. (2013). From Sequence and Forces to Structure, Function, and Evolution of Intrinsically Disordered Proteins. Structure.

[28] Herren A.W., Bers D.M., Grandi E. (2013). Post-translational modifications of the cardiac Na channel: Contribution of CaMKII-dependent phosphorylation to acquired arrhythmias. Am. J. Physiol. Heart Circ. Physiol..

[29] Schuch J.B., Paixão-Côrtes V.R., Friedrich D.C., Tovo-Rodrigues L. (2016). The contribution of protein intrinsic disorder to understand the role of genetic variants uncovered by autism spectrum disorders exome studies. Am. J. Med. Genet. Part. B Neuropsychiatr. Genet..

[30] Uversky V.N., Oldfield C.J., Dunker A.K. (2008). Intrinsically Disordered Proteins in Human Diseases: Introducing the D^2^ Concept. Annu. Rev. Biophys..

[31] Vacic V., Markwick P.R., Oldfield C.J., Zhao X., Haynes C., Uversky V.N., Iakoucheva L.M. (2012). Disease-associated mutations disrupt functionally important regions of intrinsic protein disorder. PLoS Comput. Biol..

[32] Tsang B., Pritisanac I., Scherer S.W., Moses A.M., Forman-Kay J.D. (2020). Phase Separation as a Missing Mechanism for Interpretation of Disease Mutations. Cell.

[33] Cheng J., Tester D.J., Tan B.H., Valdivia C.R., Kroboth S., Ye B., January C.T., Ackerman M.J., Makielski J.C. (2011). The common African American polymorphism SCN5A-S1103Y interacts with mutation SCN5A-R680H to increase late Na current. Physiol. Genom..

[34] Gando I., Morganstein J., Jana K., McDonald T.V., Tang Y., Coetzee W.A. (2017). Infant sudden death: Mutations responsible for impaired Nav1.5 channel trafficking and function. Pacing Clin. Electrophysiol..

[35] Nathan S., Gabelli S.B., Yoder J.B., Srinivasan L., Aldrich R.W., Tomaselli G.F., Ben-Johny M., Amzel L.M. (2021). Structural basis of cytoplasmic NaV1.5 and NaV1.4 regulation. J. Gen. Physiol..

[36] Zeppenfeld K., Tfelt-Hansen J., de Riva M., Winkel B.G., Behr E.R., Blom N.A., Charron P., Corrado D., Dagres N., de Chillou C. (2022). 2022 ESC Guidelines for the management of patients with ventricular arrhythmias and the prevention of sudden cardiac death. Eur. Heart. J..

[37] Richards S., Aziz N., Bale S., Bick D., Das S., Gastier-Foster J., Grody W.W., Hegde M., Lyon E., Spector E. (2015). Standards and guidelines for the interpretation of sequence variants: A joint consensus recommendation of the American College of Medical Genetics and Genomics and the Association for Molecular Pathology. Genet. Med..

[38] Pappone C., Brugada J., Vicedomini G., Ciconte G., Manguso F., Saviano M., Vitale R., Cuko A., Giannelli L., Calovic Z. (2017). Electrical Substrate Elimination in 135 Consecutive Patients With Brugada Syndrome. Circ. Arrhythm. Electrophysiol..

[39] Pappone C., Ciconte G., Manguso F., Vicedomini G., Mecarocci V., Conti M., Giannelli L., Pozzi P., Borrelli V., Menicanti L. (2018). Assessing the Malignant Ventricular Arrhythmic Substrate in Patients With Brugada Syndrome. J. Am. Coll. Cardiol..

