# Supplementary material for: Challenges in Brugada Syndrome Stratification: Investigating SCN5A Mutation Localization and Clinical Phenotypes
Source: Int J Mol Sci. 2023 Nov 23;24(23):16658. doi: 10.3390/ijms242316658 (PMC10706434; doi:10.3390/ijms242316658)

Supplemental Appendix

Challenges in Brugada Syndrome Stratification: Investigating SCN5A Mutation Localization and Clinical Phenotypes

Adriana Tarantino[1,2], Giuseppe Ciconte[2,3], Andrea Ghiroldi[1], Flavio Mastrocinque[3], Emanuele Micaglio [3], Antonio Boccellino [3], Gabriele Negro [3], Marco Piccoli [1], Federica Cirillo [1], Gabriele Vicedomini[3], Vincenzo Santinelli[3], Luigi Anastasia [1,2,*], and Carlo Pappone [2,3,*]

[1]Institute for Molecular and Translational Cardiology (IMTC), IRCCS Policlinico San Donato, piazza Malan 2, 20097, San Donato Milanese, Milan, Italy;
[2]School of Medicine, University Vita-Salute San Raffaele, via Olgettina 58, 20132, Milan, Italy;
[3]Arrhythmology Department, IRCCS Policlinico San Donato, piazza Malan 2, 20097, San Donato Milanese, Milan, Italy

Index

Supplemental Table S1	Page S2
Supplemental Table S2	Page S5
Supplemental Table S3	Page S6
Supplemental Figure S1	Page S7
Supplemental Figure S2	Page S8

Supplemental Table S1. Substrate areas and potential durations in 36 probands at high risk for sudden cardiac death.

	Amino acid change	IDL	Mutation Type	Mutation Impact	Substrate size Baseline (cm^2)	Substrate size after Ajmaline (cm^2)	Potential duration Baseline (ms)	Potential duration after Ajmaline (ms)
c.481G>A	p.Glu161Lys	NO	DAM	Missense	9	19	98	177
	p.Arg222Gln	NO	DAM	Missense	17	24	174	285
c.4140C>G	p.Asn1380Lys	YES	DAM	Missense	7	24	158	198
c.2414C>T	p.Ser805Leu	NO	BEN	Missense	9	18	144	244
c.3917G>A	p.Arg1306His	NO	DAM	Missense	11	18	166	253
c.5129C>T	p.Ser1710Leu	NO	DAM	Missense	12	19	189	224
c.2182G>A	Val728Ile	NO	BEN	Missense	10	17	110	249
c.3917G>A	p.Arg1306His	NO	DAM	Missense	14	24	185	277
	p.Arg222Phe	NO	BEN	Missense	15	32	185	282
c.2893C>T	Arg965Cys	NO	DAM	Missense	9	24	77	225
c.2314G>A	p.Asp772Asn	YES	DAM	Missense	0	6	70	215

c.4813G>T	p.Gly1605Cys	NO	DAM	Missense	11	18	169	242
c.4534C>T	p.Arg1512Trp	YES	DAM	Missense	4	17	104	196
c.4057G>A	p.Val1353Met	NO	DAM	Missense	8	18	155	203
c.1673A>G	p.Glu453Asp	YES	BEN	Missense	0	5	97	250
c.692C>T	p.Ser231Leu	NO	BEN	Missense	9	24	112	241
c.5000T>A	p.Val1667Asp	NO	DAM	Missense	12	19	94	205
c.4501C>G	p.Leu1501Val	YES	DAM	Missense	8	18	78	184
c.1144C>T	Leu382Phe	NO	DAM	Missense	4	18	211	211
c.2441G>A	p.Arg814Gln	NO	DAM	Missense	7	35	72	239
c.1045G>A	Asp349Asn	NO	DAM	Missense	11	15	89	200
c.3673G>A	p.Glu1225Lys	NO	DAM	Missense	9	20	92	202
c.2542A>G	p.Ile848Val	NO	DAM	Missense	0	13	63	219
c.733C>A	p.Gln245Lys	NO	DAM	Missense	6	16	147	216

c.4720G>A	p.Glu1574Lys	NO	DAM	Missense	8	20	150	224
c.655C>T	Arg219Cys	NO	DAM	Missense	11	16	96	215
c.1041C>A	p.Asn347Lys	NO	DAM	Missense	6	18	82	253
c.5102T>G	p.Met1701Arg	NO	DAM	Missense	11	24	191	283
c.589G>T	p.Asp197Tyr	NO	DAM	Missense	8	13	179	250
c.3917G>A	p.Arg1306His	NO	DAM	Missense	5	11	160	257
c.422T>A	p.Ile141Asn	NO	DAM	Missense	10	30	87	215
c.4894C>T	p.Arg1632Cys	NO	DAM	Missense	9	25	199	215
c.1890G>A	p.Thr630=	YES	BEN	Missense	12	20	93	215
P1506T	p.Pro1506Thr	YES	DAM	Missense	10	20	165	219
c.5494C>G	p.Gln1832Glu	YES	BEN	Missense	0	13,8	0	212
c.2039G>A	p.Arg680His	YES	BEN	Missense	0	35,1	0	241

BEN: benign; DAM:damaging

Supplemental Table S2. Clinical characteristics of the 36 probands at high risk for sudden cardi ac death with mutations in IDL domain described as "benign" or "damaging"

	***SCN5A* mutations in IDL - BENIGN 4 (45%)**	***SCN5A* mutations IDL - DAMAGING 5 (55%)**	**P value**
**Spontaneous type 1 pattern, n (%)**	2(50)	1(20)	0.3427
**Family history of SD, n (%)**	2(50)	2(40)	0.7641
**Aborted Cardiac Arrest, n (%)**	0	0	#
**Syncope, n (%)**	4 (100)	3(60)	0.4901
**Spontaneous VT/VF requiring ICD therapy, n (%)**	2(50)	0	0.3427
Atrial Fibrillation, n (%)	4(100)	3(60)	0.4444
Atrial Flutter, n (%)	0	0	#

Supplemental Table S3 Clinical characteristics of the 36 probands at high risk for sudden cardi ac death with mutations in TD described as "benign" or "damaging"

	**SCN5A mutations in TD - BENIGN 4 (15%)**	**SCN5A mutations in TD - DAMAGING 23 (85%)**	**P value**
**Spontaneous type 1 pattern, n (%)**	1(25)	7(28)	0.8261
**Family history of SD, n (%)**	1(25)	5(22)	0.2561
**Aborted Cardiac Arrest, n (%)**	3(75)	6(26)	0.0554
**Syncope, n (%)**	0	10(43)	0.2638
**Spontaneous VT/VF requiring ICD therapy, n (%)**	1(25)	12(52)	0.3154
Atrial Fibrillation, n (%)	1(25)	8(35)	0.7016
Atrial Flutter, n (%)	0	1(4)	1

Supplemental Figure S1. PONDR disordered regions prediction among NaVs

.

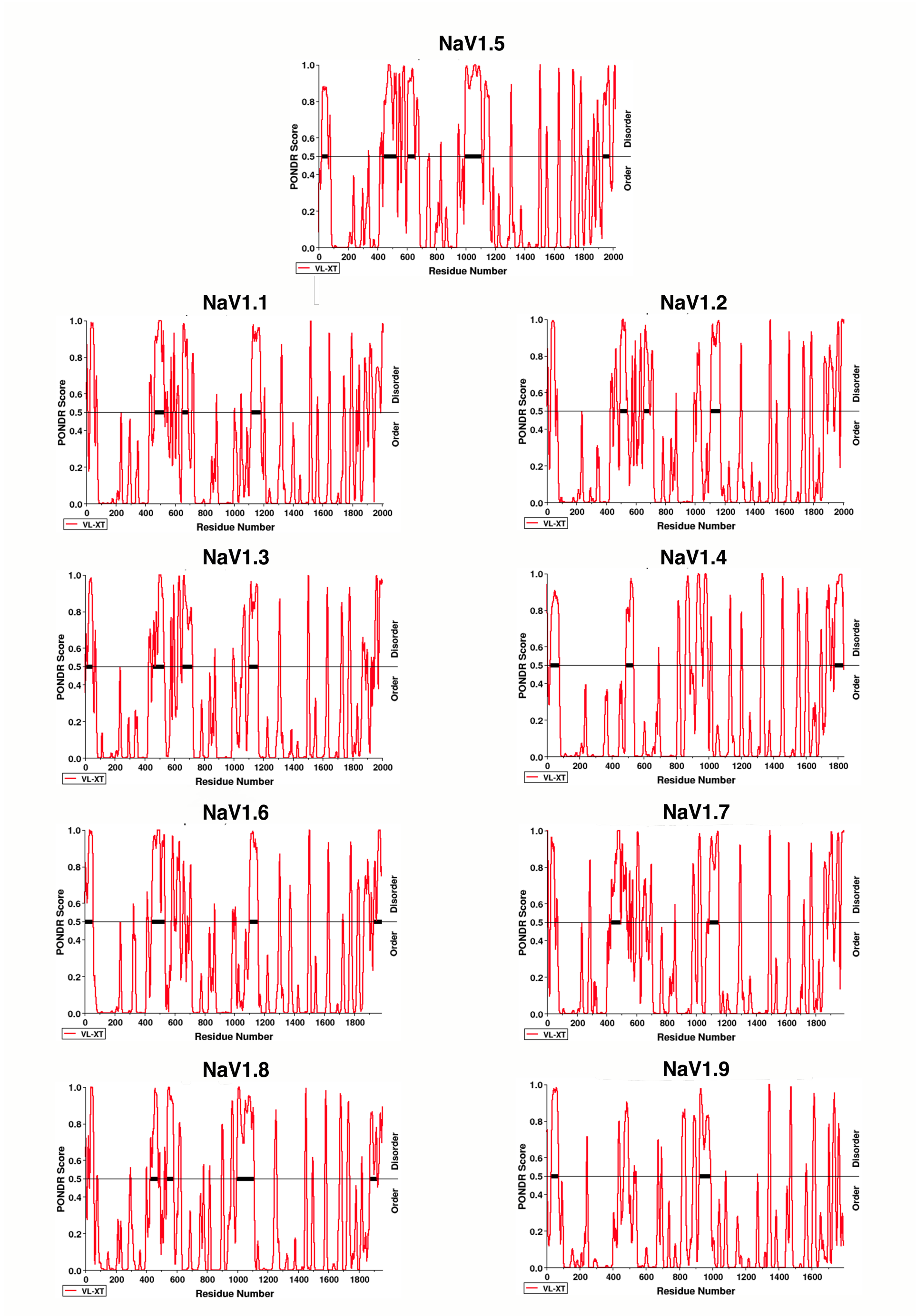

Supplemental Figure S2. Alignment of NaV IDR regions among family members. Clustal Omega Aligment of NaV1.1- P35498, NaV1.2- Q99250, NaV1.3- Q9NY46, NaV1.4- P35499, Na V1.5- Q14524, NaV1.6, NaV1.7- Q01118, NaV1.8- Q9UQD0, NaV1.9- Q9Y5Y9 in: D2 (pos .718-938),IDR1(pos.20- 66);IDR2(pos.441-532);IDR3(pos.993-1111—1125-1162);IDR4 (pos.1932-1980).

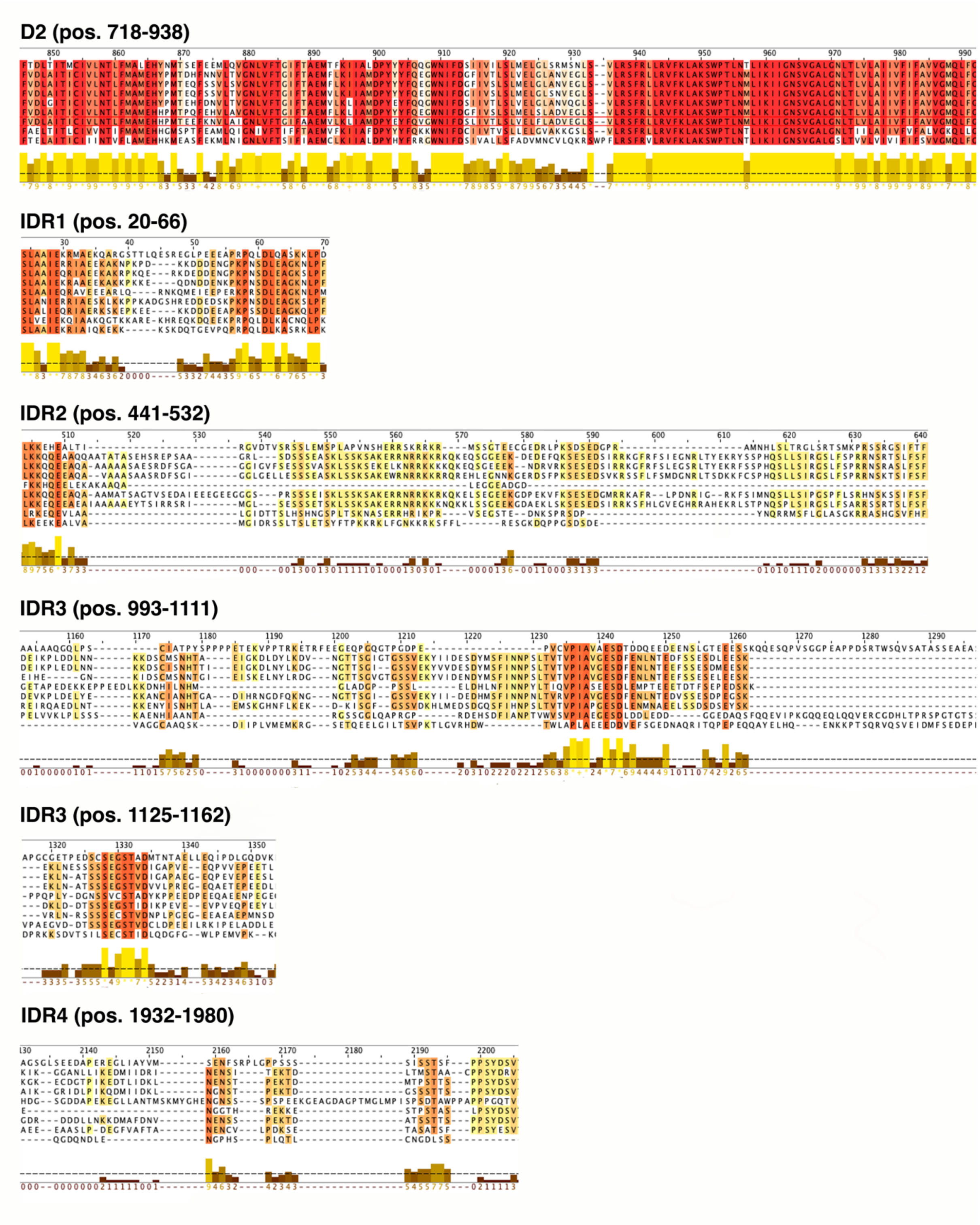

Supplement: Supplementary file 1 [file ijms-24-16658-s001.zip › ijms-2688384-supplementary.pdf]
